# Risk of intradialytic hypotension among different antihypertensives in haemodialysis patients

**DOI:** 10.1093/ckj/sfaf159

**Published:** 2025-05-23

**Authors:** Tzu-Ying Chen, Miyuki Hsing-Chun Hsieh, Fang-Yi Su, Chung-Yi Li, Ming-Cheng Wang, Chin-Chung Tseng, Kuan-Hung Liu, Jung-Hsien Chiang, Edward Chia-Cheng Lai

**Affiliations:** School of pharmacy, Institute of Clinical Pharmacy and Pharmaceutical Sciences, College of Medicine, National Cheng Kung University, Tainan, Taiwan; Department of Pharmacy Practice, Tri-Service General Hospital, Taipei, Taiwan; School of pharmacy, Institute of Clinical Pharmacy and Pharmaceutical Sciences, College of Medicine, National Cheng Kung University, Tainan, Taiwan; Population Health Data Centre, National Cheng Kung University, Tainan, Taiwan; Department of Computer Science and Information Engineering, National Cheng Kung University, Tainan, Taiwan; Department of Public Health, College of Medicine, National Cheng Kung University, Tainan, Taiwan; Department of Public Health, College of Public Health, China Medical University, Taichung, Taiwan; Department of Healthcare Administration, College of Medical and Health Science, Asia University, Taichung, Taiwan; Division of Nephrology, Department of Internal Medicine, National Cheng Kung University Hospital, College of Medicine, National Cheng Kung University, Tainan, Taiwan; Division of Nephrology, Department of Internal Medicine, National Cheng Kung University Hospital, College of Medicine, National Cheng Kung University, Tainan, Taiwan; Division of Nephrology, Department of Internal Medicine, National Cheng Kung University Hospital, College of Medicine, National Cheng Kung University, Tainan, Taiwan; Institute of Clinical Medicine, College of Medicine, National Cheng Kung University, Tainan, Taiwan; Department of Computer Science and Information Engineering, National Cheng Kung University, Tainan, Taiwan; School of pharmacy, Institute of Clinical Pharmacy and Pharmaceutical Sciences, College of Medicine, National Cheng Kung University, Tainan, Taiwan; Population Health Data Centre, National Cheng Kung University, Tainan, Taiwan

**Keywords:** antihypertensive drugs, haemodialysis, intradialytic hypotension

## Abstract

**Background:**

Evaluating the impact of antihypertensives on intradialytic hypotension (IDH) risk is crucial, given their frequent use in haemodialysis (HD) patients. This study assessed the comparative safety of various antihypertensives in relation to IDH.

**Methods:**

This retrospective cohort study at a tertiary medical centre in Taiwan included adult patients initiating HD from 2016 to 2021. Using electronic health records, we retrieved patient demographics and data covering the first 3 years of HD. We classified antihypertensives as angiotensin-converting enzyme inhibitors (ACEIs)/angiotensin receptor blockers (ARBs), alpha blockers, beta blockers and dihydropyridine calcium channel blockers (DHP-CCBs). We defined IDH as a nadir intradialytic blood pressure decrease to <90/100 mmHg (Nadir 90/100) or a decrease of 40 mmHg (Fall 40). Using a generalized linear mixed model, we analysed odds ratios (ORs) and 95% confidence intervals (CIs) for IDH.

**Results:**

This study included 115 patients, covering 39 371 HD sessions over an average follow-up of 27.5 months. We identified 4534 IDH events based on Nadir 90/100, with event rates of 5.0% for ACEIs/ARBs, 3.0% for alpha blockers, 6.7% for beta blockers and 4.0% for DHP-CCBs. For Fall 40, we found 4814 IDH events, with rates of 11.5%, 7.7%, 11.4% and 9.2%, respectively. ACEIs/ARBs and beta blockers did not significantly alter IDH risk. CCBs and alpha blockers significantly reduced IDH risk, with Nadir 90/100–adjusted ORs of 0.65 (95% CI 0.55–0.76) for CCBs and 0.57 (95% CI 0.43–0.74) for alpha blockers, compared with sessions without these medications. For Fall 40, adjusted ORs were 0.73 (95% CI 0.64–0.83) for CCBs and 0.61 (95% CI 0.49–0.74) for alpha blockers.

**Conclusions:**

Use of beta blockers and ACEIs/ARBs had a neutral effect on IDH risk, while DHP-CCBs and alpha blockers were associated with lower risks of IDH and might be considered for patients with IDH risk factors. These findings support the safe use of antihypertensives in HD patients, offering valuable guidance for clinical medication adjustments.

## Graphical Abstract

**Graphical Abstract:**
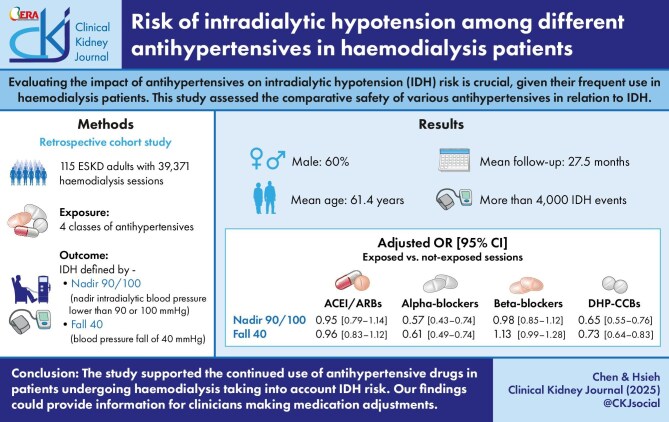

KEY LEARNING POINTS **What was known:**

• Intradialytic hypotension (IDH) affects 5–30% of haemodialysis (HD) sessions and can lead to severe complications, including organ ischaemia, heart failure and increased mortality.
• Literature suggests varying risks of IDH with different antihypertensives, but evidence remains limited and inconsistent.

**This study adds:**

• This is the first study to systematically compare the impact of different antihypertensive drug classes on IDH.
• Our findings show that angiotensin-converting enzyme inhibitors (ACEIs)/angiotensin receptor blockers and beta blockers have neutral effects on IDH risk, while alpha blockers and dihydropyridine calcium channel blockers (DHP-CCBs) are associated with a lower IDH risk.

**Potential impact:**

• Our study supports the use of ACEIs/ARBs and beta blockers without increasing IDH risk and suggests that alpha blockers and DHP-CCBs may be beneficial in preventing IDH. These findings provide valuable guidance for clinicians in managing antihypertensive therapy in HD patients.

## INTRODUCTION

Intradialytic hypotension (IDH) occurs frequently during haemodialysis (HD), affecting 5–30% of all treatments [1–3]. This can lead to unfavourable clinical outcomes, such as organ ischaemia, heart failure and access-related complications [4, 5]. Additionally, IDH increases the risk of cardiovascular accidents and overall mortality in HD patients [6, 7].

While antihypertensive drugs like angiotensin-converting enzyme inhibitors (ACEIs)/angiotensin receptor blockers (ARBs), beta blockers, calcium channel blockers (CCBs) and alpha blockers are commonly prescribed for HD patients [8], their effect with regard to IDH can vary, due to their distinct mechanisms and influence on haemodynamics [9–12]. However, previous studies investigating the impact of antihypertensive drugs on IDH have been notably sparse, leading to a lack of clear and strong recommendations in clinical guidelines [13]. Antihypertensive medications are thought to increase the risk of IDH, thus patients are often advised to withhold these drugs before dialysis [14]. This practice, which is not supported by strong evidence, could result in intradialytic hypertension and poorly controlled blood pressure (BP) [15].

A limited number of studies have prioritized IDH in their research, with many considering it merely a secondary outcome. Consequently, the studies may not have the statistical robustness to detect significant differences between treatment groups [3, 16–21]. In addition, previous prospective studies only compared single agents to a placebo rather than comparing different classes of antihypertensive drugs [16–21], limiting their clinical applicability. Furthermore, the definitions of antihypertensive drug exposure and IDH have been inconsistent, complicating the comparison and interpretation of research findings [22–25].

The definitions used in this study, Nadir 90/100 [intradialytic systolic BP (SBP) decreasing to <90 mmHg, or <100 mmHg for patients with a pre-intradialytic BP ≥160 mmHg) and Fall 40 (a decrease in SBP by ≥40 mmHg during HD) have been shown to be correlated with increased mortality risk [7, 26], yet they have not been widely applied in past IDH research, drawing into question the relevance of such research to patient prognosis. Moreover, the majority of previous studies did not focus on new dialysis patients, potentially introducing survivor bias, as the risk of IDH may increase with a longer duration of HD treatment.

To address the aforementioned limitations, we employed precise drug exposure and outcome definitions in this study. By integrating a design that included new dialysis patients and using pragmatic comparators, we expected to provide evidence concerning the comparative effects of different antihypertensive drugs on IDH, hypothesizing that IDH risk varies across these drug classes.

## MATERIALS AND METHODS

### Data source and study population

Data were obtained through an extensive review of electronic medical records by nephrologists from National Cheng Kung University Hospital, a premier tertiary medical centre in Taiwan.

This study is a retrospective cohort study and included 182 patients who underwent HD at National Cheng Kung University Hospital during the study period (February 2016–October 2021). All patients underwent conventional HD, defined specifically as HD (excluding haemodiafiltration, sustained low-efficiency dialysis or other modalities) performed three times per week with a target session duration of 4 h. We identified new dialysis patients who were >20 years of age at the time of dialysis initiation and had received at least one prescription for any class of antihypertensive drugs during the entire study period. A 1-month look-back period was applied to ascertain new dialysis patients. We included dialysis sessions that occurred within 3 years of HD initiation. Furthermore, we excluded sessions during the initial 90-day period after dialysis initiation for each patient, as this adjustment phase is marked by clinical instability and frequent modifications to fluid removal targets to determine optimal dry weight, often resulting in missing dry weight data. Exclusion of these sessions minimizes variability, improves data completeness and ensures a more reliable analysis of stable patient conditions. The study was approved by the Institutional Review Board of National Cheng Kung University Hospital (A-ER-110-327) and adhered to the principles of the Declaration of Helsinki. Patient informed consent was waived due to the retrospective nature of the study and the absence of additional harm to the included patients.

### Exposures and follow-up

The exposures of interest were the four classes of antihypertensive drugs recommended by clinical guidelines as the primary choices for antihypertensive agents in the dialysis population: ACEIs/ARBs, alpha blockers, beta blockers and dihydropyridine calcium channel blockers (DHP-CCBs) [13, 27, 28]. Since our unit of observation was individual dialysis sessions, we identified the types of antihypertensive medications to which patients were exposed on the day of each session. The medications included in the exposure definition are listed in Supplemental Table 1. Specifically, for alpha blockers, we only included non-selective α-1 blockers such as doxazosin and terazosin, as these agents are primarily indicated for hypertension. All patients were followed from the start to the end of each session, which lasted approximately 4 h.

### Outcome of interest

In the National Cheng Kung University Hospital, strict protocols were in place for monitoring patients’ BP during HD sessions. Within our database, comprehensive dialysis records were accessible, including SBP measurements obtained immediately before the initiation of each dialysis session, at hourly intervals throughout the duration of dialysis and after dialysis treatment. The outcome of interest was the occurrence of IDH, assessed during each dialysis session using two definitions, Nadir 90/100 and Fall 40, which have been shown to correlate more strongly with mortality, thus providing a clinically relevant prognosis for dialysis patients [7, 26]. Nadir 90/100 was defined as the lowest intradialytic SBP decreasing to <90 mmHg, or <100 mmHg for those with a pre-dialysis SBP >160 mmHg. Fall 40 was defined as a decrease in SBP of ≥40 mmHg during dialysis. BPs were measured at the start of dialysis, every hour during the session and after treatment.

### Covariates

We collected patient characteristics by reviewing medical records from at least 6 months prior to the HD session. The covariates included demographic data such as age, sex, HD vintage and history of coronary artery disease (CAD), diabetes mellitus (DM), hypertension (HTN) and left ventricular ejection fraction (LVEF). LVEF was documented based on the echocardiogram performed closest to each dialysis session. More than 93% of the included sessions had LVEF records, with a median time of recording within 3 months, which we believe reasonably reflects the patient's current cardiac function. Additionally, CAD, DM and HTN were considered present if the patient had corresponding records documented during the study period. We also documented dialysis-related variables, including dry weight, post-dialysis body weight, ultrafiltration rate (UFR), ultrafiltration:dry weight ratio, dialysate flow rate, blood flow, dialysate calcium, average dialysate temperature, normal saline supplementation and pre- and post-HD systolic and diastolic BP. Additionally, we recorded co-medications, such as vasodilators, midodrin, and erythropoiesis-stimulating agents (ESAs); vasodilators and midodrine were included if prescribed on dialysis days and ESAs if prescribed within a 7-day window of each session (see Supplementary Table 2).

### Statistical analyses

To analyse the effect of different classes of antihypertensive drugs on the risk of IDH, we applied a generalized linear mixed effects model as the inferential analysis to account for data dependency within an individual. Antihypertensive exposures were evaluated using fixed effects while individuals were treated as random effects. The fixed effects related to the effects of different types of antihypertensive drugs, while the random effects accounted for the variability of values between different patients. We modelled our data using a binomial distribution with a logit link function. This modelling approach permitted us to calculate the log odds of an IDH event occurring in association with our variables of interest. In addition, we applied an unstructured covariance matrix as the working correlation matrix. We generated odds ratios (ORs) with 95% confidence intervals (CIs), which estimated the odds of IDH occurrence in association with exposure versus non-exposure to a particular class of antihypertensive drugs, assuming all other variables were held constant. For example, in the comparison of ACEI versus non-ACEI sessions, the numerator included all sessions exposed to ACEIs, while the denominator included all other HD sessions, including those with exposure to other classes of antihypertensives and those without any antihypertensive treatment. To account for the combined use of these drugs, the exposure status for each antihypertensive class was represented using four binary variables in the regression model.

We controlled for potential confounding by adjusting for various covariates stated in the previous paragraph, including comorbidities, the ultrafiltration:dry weight ratio, dialysate flow rate, dialysate calcium concentration, dialysate average temperature, pre-HD SBP and co-medications. We further controlled for environmental factors by adjusting for average ambient temperature since evidence has shown that BP, the risk of IDH and the effect of some medications might be influenced by variations in ambient temperature [29–32].

We conducted sensitivity analyses by adjusting the definitions of exposure and outcome. Antihypertensive drug exposure was redefined as continuous use for at least 3 months prior to a given dialysis session, as studies suggest that antihypertensive drugs may influence the risk of IDH through the reversal of left ventricular hypertrophy (LVH), a process that requires long-term exposure [33–35]. Additionally, IDH requiring intervention has been used as a criterion for defining IDH in various guidelines [22–25]. Therefore, we expanded our outcome definitions beyond the original measures of intradialytic BP (Nadir 90/100 and Fall 40) to include the requirement of clinical management, which included supplemental administration of normal saline or failure to achieve the target dry weight (see Supplementary Table 4). Analyses were performed using SAS version 9.4 (SAS Institute, Cary, NC, USA). For all statistical tests, two-sided *P*-values <0.05 were considered statistically significant.

## RESULTS

### Characteristics of the study cohort

**Figure 1:**
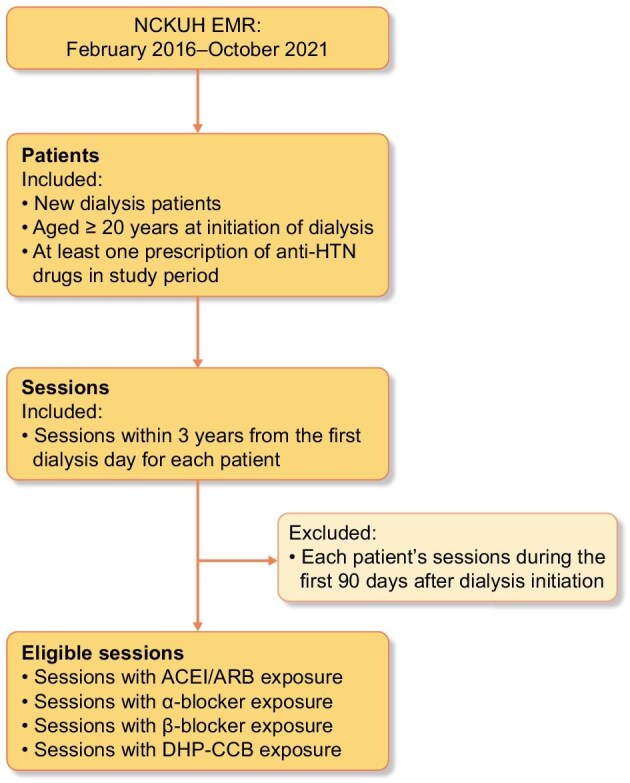
Cohort selection flow chart. Flow chart illustrates the selection process for HD sessions included in the study. A total of 39 371 eligible sessions were assessed for exposure to four classes of antihypertensive drugs: ACEIs/ARBs, alpha blockers, beta blockers and DHP-CCBs. It was possible for a session to have exposure status for multiple classes of antihypertensive drugs.

A total of 182 patients underwent HD between 2016 and 2021. After excluding 67 patients who had already been receiving HD prior to the study period and were therefore not initiators, 115 patients were included in the analysis, accounting for a total of 39 371 HD sessions (Fig. 1). The mean duration of included HD was 27.5 ± 8.7 months and the mean age was 61.4 ± 15.1, with 60% being male. Of all patients, 67.8%, 38.3%, 68.7% and 83.5% had been exposed to ACEIs/ARBs, alpha blockers, beta blockers and DHP-CCBs, respectively. Of all sessions, 23.7%, 8.5%, 31.7% and 33.6% involved ACEIs/ARBs, alpha blockers, beta blockers and DHP-CCBs, respectively, while 49% did not involve any of the aforementioned drugs. Hypertension was the most common comorbidity among the patients. Dialysis-related variables were largely consistent across all sessions. The average pre-HD SBP was 136 mmHg for all sessions, with slightly higher values observed in sessions where drugs were administered. Detailed characteristics by exposure to antihypertensive drugs are provided in Table 1.

**Table 1:** Baseline characteristics of included HD sessions.

		Antihypertensive drugs exposed				
Characteristics	Overall	ACEI/ARB	Alpha blocker	Beta blocker	DHP-CCB	No antihypertensive treatment
HD sessions, *n* (%)	39 371 (100)	9334 (24)	3347 (9)	12 352 (31)	13 236 (34)	19 207 (49)
Patients, *n* (%)^b^	115 (100)	78 (68)	44 (38)	79 (69)	96 (84)	105 (91)
Duration of included HD (days), mean (SD)	825.2 (261.1)	813.5 (258.4)	759.5 (278.0)	838.6 (244.3)	825.6 (254.4)	847.3 (246.2)
Demographic data						
Age (years), mean (SD)	61.4 (15.1)	60.2 (15.7)	58.9 (15.7)	59.9 (14.8)	61 (15.4)	62.6 (15.4)
Male, *n* (%)	69 (60)	51 (65)	32 (73)	46 (58)	60 (63)	61 (58)
CAD, *n* (%)	28 (24)	19 (24)	8 (18)	18 (23)	18 (19)	27 (26)
Diabetes mellitus, *n* (%)	45 (39)	32 (41)	14 (32)	29 (37)	38 (40)	42 (40)
Hypertension, *n* (%)	90 (78)	64 (82)	38 (86)	67 (85)	78 (81)	81 (77)
LVEF, mean (SD)	64.1 (11.8)	62.4 (11.4)	64.7 (10.6)	63.8 (11.3)	65.5 (10.4)	64.3 (11.9)
Dialysis-related variables^a^						
Dry weight (kg), mean (SD)	59.6 (13.6)	61.4 (15.7)	64 (14.6)	62.9 (16.1)	59.6 (14.3)	58.7 (11.5)
Post-HD body weight (kg), mean (SD)	61.7 (14.0)	63.5 (16.5)	65.2 (15.0)	64.2 (16.3)	61.3 (15.0)	61.1 (11.7)
Ultrafiltration (kg), mean (SD)	2.3 (1.2)	2.3 (1.1)	2.2 (1.1)	2.4 (1.2)	2.3 (1.1)	2.4 (1.2)
UF/DW (%), mean (SD)	4 (1.8)	3.8 (1.8)	3.5 (1.6)	3.8 (1.7)	3.9 (1.7)	4.1 (1.9)
Blood flow (ml/min), mean (SD)	269.1 (31.6)	278.2 (32.3)	289.8 (31.4)	278.3 (32.5)	276.2 (33.3)	263.0 (28.8)
Dialysate flow (ml/min), mean (SD)	585.4 (129.0)	627.8 (140.9)	666.5 (138.2)	614.4 (135.6)	604.4 (136.4)	568.3 (121.2)
Dialysate average temperature (°C), mean (SD)	36.1 (0.4)	36.1 (0.3)	36.1 (0.4)	36.2 (0.4)	36.2 (0.4)	36.1 (0.3)
Normal saline (ml), mean (SD)	513.8 (69.2)	523.9 (96.1)	540.7 (131.4)	524.3 (90.5)	518.3 (83.7)	509.6 (55.9)
Pre-HD SBP (mmHg), mean (SD)^a^	136 (22.4)	143.7 (21.3)	145.5 (19.4)	140.6 (21.5)	143.8 (19.9)	130.4 (22.3)
Pre-HD DBP (mmHg), mean (SD)	72.9 (14.4)	76.9 (15.0)	75.1 (14.8)	74.5 (14.3)	76.4 (14.1)	70.6 (14.4)
Post-HD SBP (mmHg), mean (SD)	131.5 (28.6)	144.4 (25.4)	150 (25.0)	139 (26.2)	143.6 (25.0)	123.1 (28.8)
Post-HD DBP (mmHg), mean (SD)	73.1 (17.0	79.8 (19.1)	78.8 (16.6)	75.3 (15.6)	78.6 (17.2)	69.7 (15.0)
Dialysate calcium (mEq/l), *n* (%)						
2.5	17 061 (43)	3598 (39)	1721 (51)	4380 (36)	6069 (46)	8274 (43)
3	18 865 (48)	4545 (49)	960 (29)	6359 (52)	5666 (43)	9652 (50)
3.5	3082 (8)	1163 (13)	666 (20)	1522 (12)	1429 (11)	1053 (5)
Co-medication, *n* (%)^a^						
Midodrine	8715 (22)	879 (9)	79 (2)	2044 (17)	716 (5)	6003 (31)
Vasodilator	2235 (6)	940 (10)	431 (13)	1122 (9)	1551 (12)	304 (2)
Erythropoietin	33 271 (85)	8462 (91)	3181 (95)	10 633 (86)	11 866 (90)	15 662 (82)
Average ambient temperature (°C), mean (SD)	25.1 (4.3)	25.1 (4.4)	25.2 (4.5)	25.1 (4.4)	24.8 (4.4)	25.1 (4.2)

a Characteristics are presented in sessions.

UF/DW: ultrafiltration divided by dry weight; DBP: diastolic BP; SD: standard deviation.

### Event rates of IDH

Across all the included HD sessions, we identified a total of 4534 IDH events as defined by Nadir 90/100, contributing to an overall event rate of 11.5%. IDH event rates were 5.0%, 3.0%, 6.7% and 4.0% for the sessions involving ACEIs/ARBs, alpha blockers, beta blockers and DHP-CCBs, respectively. We identified a total of 4814 IDH events as defined by Fall 40, resulting in an overall event rate of 12.2%. IDH event rates for the sessions involving ACEIs/ARBs, alpha blockers, beta blockers and DHP-CCBs were 11.5%, 7.7%, 11.4% and 9.2%, respectively.

### ORs of IDH across classes of antihypertensive drugs

**Figure 2:**
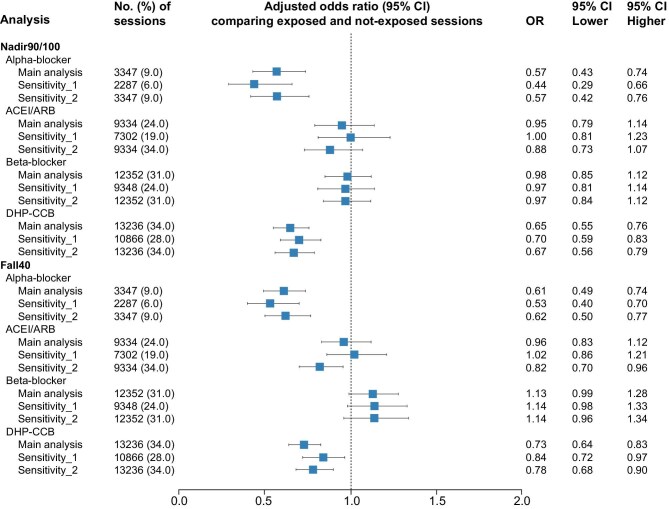
ORs of IDH for different antihypertensive drug classes. Forest plot displays the adjusted ORs with 95% CIs for the risk of IDH associated with exposure to different antihypertensive drug classes compared with not-exposed sessions. The plot includes the main analysis and two sensitivity analyses for each drug class under the definitions of Nadir 90/100 and Fall 40. Sensitivity_1 represents the analysis with an altered definition of drug exposure, while Sensitivity_2 represents the analysis with an altered definition of the IDH outcome. The horizontal lines represent the 95% CIs for each OR and the squares indicate the point estimate of the OR. The vertical line corresponds to an OR of 1.0, indicating no effect.

The estimated ORs across different antihypertensive drug classes are presented in Table 2 and illustrated in Fig. 2 When IDH was defined by Nadir 90/100 and sessions with DHP-CCBs exposure were compared with those with no exposure, the adjusted OR (aOR) was 0.65 (95% CI 0.55–0.76), indicating a decrease in the odds of IDH occurrence. A similar reduction was observed for alpha blockers, with an aOR of 0.57 (95% CI 0.43–0.74). For ACEIs/ARBs [aOR 0.95 (95% CI 0.79–1.14)] and beta-blockers [aOR 0.98 (95% CI 0.85–1.12)], no significant effects were found with regard to IDH occurrence. Similar results were observed when the outcome was defined by Fall 40. Compared with sessions with no drug given, sessions using DHP-CCBs [aOR 0.73 (95% CI 0.64–0.83)] and alpha blockers [aOR 0.61 (95% CI 0.49–0.74)] were significantly associated with lower odds of IDH events. In contrast, the effects of ACEIs/ARBs and beta blockers were neutral.

**Table 2:** ORs of IDH among different classes of antihypertensive drugs.

		Nadir 90/100		Fall 40	
Drug class	Sessions	Events	OR (95% CI)^a^	Events	OR (95% CI)^a^
ACEI/ARB^a^	9334	468	0.95 (0.79–1.14)	1070	0.96 (0.83–1.12)
Alpha blocker	3347	102	0.57 (0.43–0.74)	257	0.61 (0.49–0.74)
Beta blocker	12 352	822	0.98 (0.85–1.12)	1409	1.13 (0.99–1.28)
DHP-CCB	13 236	531	0.65 (0.55–0.76)	1214	0.73 (0.64–0.83)

a Adjusted for sex, age, diabetes mellitus, hypertension, CAD, LVEF, pre-HD SBP, dialysate flow rate, blood flow, dialysate calcium, ultrafiltration:dry weight ratio, co-medications and average ambient temperature.

### Results of sensitivity analyses

The characteristics of patients and sessions included in the sensitivity analyses with altered exposure definitions are shown in Supplementary Table 3. These stricter exposure definitions decreased the number of sessions involving exposure to antihypertensive drugs compared with the main analysis. Most of the parameters mirrored the main analysis. The estimated ORs of IDH among different classes of antihypertensive drugs are presented in Table 3. For IDH defined by Nadir 90/100, the finding was consistent with the main analysis. Use of DHP-CCBs or alpha blockers significantly decreased the odds of IDH events, while the effects of ACEIs/ARBs and beta blockers on IDH were neutral. As for IDH events defined by Fall 40, the results were similar.

**Table 3:** ORs of IDH among different classes of antihypertensive drugs in sensitivity analysis.

		Nadir 90/100		Fall 40	
Drug class	Sessions	Events	OR (95% CI)^a^	Events	OR (95% CI)^a^
Altered exposure definition					
ACEI/ARB	7302	350	1.00 (0.81–1.23)	806	1.02 (0.86–1.21)
Alpha blocker	2287	46	0.44 (0.29–0.66)	157	0.53 (0.40–0.70)
Beta blocker	9348	512	0.97 (0.81–1.14)	1050	1.14 (0.98–1.33)
DHP-CCB	10 866	400	0.7 (0.59–0.83)	916	0.84 (0.72–0.97)
Altered outcome definition					
ACEI/ARB	9334	410	0.88 (0.73–1.07)	872	0.82 (0.70–0.96)
Alpha blocker	3347	90	0.57 (0.42–0.76)	204	0.62 (0.50–0.77)
Beta blocker	12 352	732	0.97 (0.84–1.12)	1186	1.14 (0.96–1.34)
DHP-CCB	13 236	469	0.67 (0.56–0.79)	1024	0.78 (0.68–0.90)

a Adjusted for sex, age, diabetes mellitus, hypertension, CAD, LVEF, pre-HD SBP, dialysate flow rate, blood flow, dialysate calcium, ultrafiltration:dry weight ratio, co-medications and average ambient temperature.

After changing to a stricter outcome definition involving IDH that required intervention, the event numbers decreased to 4117 and 4156, when defined by the Nadir 90 and Fall 40 criteria, respectively. The estimated ORs are displayed in Table 3. While most of the results align with the main analysis, there was a difference regarding outcomes defined by Fall 40 and intervention. The use of ACEIs/ARBs significantly reduced the odds of clinically significant IDH.

## DISCUSSION

The association between antihypertensive drugs and the risk of IDH has not been clearly addressed in previous studies [3, 16–25]. Many believe these drugs may increase the risk of IDH [3, 13, 14]. However, our study found that ACEIs/ARBs and beta blockers, often first-line choices of antihypertensive medications for HD patients, showed neutral effects with regard to IDH. Additionally, DHP-CCBs and alpha blockers were associated with reduced risks of IDH, whether defined by Nadir 90/100 or Fall 40. These findings, consistent across sensitivity analyses, indicated that the use of different antihypertensive agents was not associated with an increased risk of IDH. In fact, the use of alpha blockers and DHP-CCBs might provide benefits in preventing IDH.

In our analysis, the use of ACEIs/ARBs did not increase the risk of IDH. Further, when the outcome was more strictly defined to consider only severe cases requiring intervention, the use of ACEIs/ARBs was found to prevent the occurrence of IDH. This finding indicated that the use of ACEIs/ARBs in patients undergoing HD is safe and reaffirmed their potential clinical benefits in cardiac remodelling and endothelial function, hence their potential to reduce the risk of IDH [36, 37]. Consistent with previous studies [17, 20], our data indicated that beta blockers did not affect IDH risk. Beta blockers, known for reducing cardiac hypertrophy, may slow heart rate, affecting fluid loss adaptation during dialysis [38]. The two drug classes are frequently prescribed to patients with CAD or heart failure, both of which could potentially influence haemodynamic responses during dialysis. However, after adjusting for a history of CAD and LVEF, no increased risk of IDH was observed with the use of ACEIs/ARBs or beta blockers. These findings indicate that both drug classes are safe for use in HD patients. Moreover, ACEIs/ARBs may offer protective effects against IDH requiring intervention, whereas beta blockers do not appear to elevate IDH risk. These results reinforce the clinical utility of these drug classes in managing cardiovascular health in HD patients without increasing the risk of IDH.

Our findings demonstrate that DHP-CCBs significantly reduce the risk of IDH, supporting previous studies. Prior research has associated DHP-CCBs with smaller decreases in intradialytic SBP [29] and a lower incidence of IDH [39]. Several mechanisms may explain the protective effect of DHP-CCBs against IDH. First, these drugs promote regression of LVH, thereby preserving cardiovascular reflexes and improving both vascular and left ventricular compliance [40, 41]. Second, long-term use of DHP-CCBs improves aortic distensibility, enhances left ventricular ejection and reduces pulse-wave velocity, an indicator of vascular stiffness, particularly in hypertensive HD patients [42]. Third, DHP-CCBs decrease oxidative stress and plasma concentrations of asymmetric dimethylarginine, a biomarker of endothelial dysfunction and cardiovascular risk in both the general population and patients with end-stage renal disease [42]. Lastly, the antihypertensive effects of DHP-CCBs are fluid-dependent, exhibiting greater efficacy in patients with increased salt and water retention [43]. Collectively, these benefits—improved left ventricular function, vascular compliance, endothelial function and fluid-dependent BP control—likely contribute to the observed reduction in IDH risk. Similarly, the reduced risk of IDH associated with alpha blockers observed in our study can be attributed to their effects on multiple IDH-related factors, including LVH, vascular stiffness and autonomic and endothelial dysfunction [9–12]. Both animal studies [44] and clinical trials [38, 45, 46] have demonstrated that alpha blockers reduce left ventricular mass and improve endothelial function, which likely contribute to their protective role against IDH. While one study suggested that alpha blockers may result in a greater reduction in SBP, this was not directly associated with an increased risk of IDH [29]. Clinically, alpha blockers are often prescribed for patients with higher baseline BP due to their potent antihypertensive effects [47]. Consistent with this, patients receiving alpha blockers in our study had the highest mean pre-HD SBP (145.5 ± 19.4 mmHg). Importantly, even after adjusting for pre-HD SBP to minimize potential confounding, alpha blockers remained significantly associated with a reduced risk of IDH.

In clinical practice, patients are often advised to withhold antihypertensive medications before dialysis, despite only limited evidence supporting this approach [14]. This practice may result in untreated hypertension, increasing the risks of mortality and cardiovascular complications [15, 48, 49]. Our findings address this issue by demonstrating that four classes of antihypertensive drugs are safe, as regards IDH risk. Given the established benefits of ACEIs/ARBs and beta blockers in reducing mortality and managing heart failure in patients with chronic kidney disease, our study supports their continued use in HD patients, regardless of IDH risk. For patients with additional IDH risk factors and no contraindications, considering DHP-CCBs or alpha blockers may be a supplementary approach where ACEIs/ARBs are insufficient or inappropriate. Our approach includes IDH outcome definitions based on both Fall 40 and Nadir 90/100. Nadir 90/100 reflects the lowest BP, relevant for patients with low baseline BP, while Fall 40 shows a significant decrease, common in those with higher pre-dialysis pressure. As these criteria capture different patient characteristics, we were able to assess a broader range of IDH risk. The results across both outcomes in our study were consistent, which indicates that the effects of antihypertensive drug classes are similar for both IDH definitions. This consistency suggests that clinical treatment strategies can be applied similarly across different BP profiles.

Our research marks a pioneering effort in assessing IDH risks with various antihypertensive drugs. As the first study to systematically compare the impact of different antihypertensive classes on IDH, it confirms the safety of these medications. It overcomes previous research limitations by focusing on IDH as the key outcome, defining drug exposures precisely and using robust data from comprehensive nephrologist-led chart reviews. By including new dialysis patients, we minimized survivorship bias, thus avoiding confounding from long-term dialysis effects. Our findings offer significant insights for clinical decision-making, enhancing IDH management in HD patients.

This study had some limitations, however, including potential misclassification of antihypertensive drug influence, as exposure was determined by the date of dialysis, possibly overlooking the need for a longer timeframe to assess drug effects. Sensitivity analyses mitigated this, with results suggesting minimal impact, likely due to the stability of antihypertensive prescriptions in HD patients. Another concern was confounding by indication, as patients on antihypertensives may have higher baseline BP or a history of heart failure, possibly confounding the association of drugs with higher IDH risks. Adjustments for pre-HD SBP and LVEF were made, showing that the use of different antihypertensive drugs did not increase IDH risks and may even have preventive effects. Additionally, by following the current protocol of hourly BP monitoring for HD patients, it is possible that we missed some IDH events that occurred between measurements. However, we believe this limitation is non-differential, affecting all groups equally. Therefore, it should not significantly impact our results, as our outcome measures were calculated using relative estimates. Furthermore, the percentage of a drug that is removed by HD may affect the probability of a drug impacting BP, and this influence was not investigated in the main analysis. To address this, we reanalysed the data after excluding sessions exposed to dialyzable antihypertensive agents—accounting for <1% in the ACEI/ARB group and up to 45% in the beta blocker group. The results for the Nadir 90/100 outcome remained consistent. For Fall 40, the findings retained the same directional trend, but the protective effect of alpha blockers became non-statistically significant, likely due to a reduced sample size. These results emphasize the importance of considering the effects of drug dialysability in future studies. Finally, as the study was conducted at a single medical centre, the patients included may have had more comorbidities and a generally more severe health status compared with those in other healthcare settings, potentially limiting the generalizability of our findings. Therefore, the effects of various antihypertensive agents on IDH in the broader HD population should be interpreted with caution.

This study revealed that the use of different antihypertensive drug classes did not increase IDH risk in patients undergoing HD. ACEIs/ARBs and beta blockers had a neutral effect on IDH incidence, supporting the safety of these medications. While DHP-CCBs and alpha blockers were associated with a potential reduction in the risk of IDH, further research is needed to confirm their effectiveness and suitability for patients with an elevated risk of IDH. These findings offer valuable insights and guidance for antihypertensive management in patients undergoing HD.

## Supplementary Material

sfaf159_Supplemental_File

## Data Availability

This data sharing extends beyond the authorization provided by the patient’s informed consent and the Institutional Review Board.
