# Supplementary material for: Risk of intradialytic hypotension among different antihypertensives in haemodialysis patients
Source: Clin Kidney J. 2025 May 23;18(6):sfaf159. doi: 10.1093/ckj/sfaf159 (PMC12166360; doi:10.1093/ckj/sfaf159)
Supplement: sfaf159_Supplemental_File [file sfaf159_supplemental_file.docx]

**Supplemental Material**

[**Supplemental Table 1:** ATC Codes of Antihypertensive Drugs Included in Study Exposure Definition (Only Listing Medications Available in NCKU Hospital) 1](#_Toc178792282)

[**Supplemental Table 2:** ATC Codes of Co-Medications Included in Baseline Characteristics (Only Listing Medications Available in NCKU Hospital) 2](#_Toc178792283)

[**Supplemental Table 3:** Characteristics of Sessions and Patients for Sensitivity Analysis Based on Exposure Definitions 3](#_Toc178792284)

[**Supplemental Table 4:** Operational Definition in Sensitivity Analysis for Outcome Definition 4](#_Toc178792285)

**Supplemental Table 1:** ATC Codes of Antihypertensive Drugs Included in Study Exposure Definition (Only Listing Medications Available in NCKU Hospital)

| Class of antihypertensive drug | | Included drugs | ATC code |
| --- | --- | --- | --- |
| **alpha-blocker** | | doxazosin, terazosin | C02CA |
| **ACEI/ARB** | **ACEI** | imidapril, captopril, ramipril, enalapril | C09AA, C09BA, C09BB, C09BX |
|  | **ARB** | losartan, candesartan, azilsartan, telmisartan, valsartan, irbesartan, olmesartan | C09CA, C09DA, C09DB, C09DX |
| **beta-blocker** | | carvedilol, labetalol, atenolol, metoprolol, bisoprolol, nebivolol, propranolol | C07 |
| **DHP-CCB** | | amlodipine, felodipine, lercanidipine, nifedipine, nicardipine | C08CA, C09DB |

**Supplemental Table 2:** ATC Codes of Co-Medications Included in Baseline Characteristics (Only Listing Medications Available in NCKU Hospital)

| Class of medication | Included drugs | ATC code |
| --- | --- | --- |
| **Vasoconstrictors** | midodrine | C01CA17 |
| **Vasodilators** | hydralazine | C02DB02 |
|  | minoxidil | C02DC01 |
|  | nitrates | C01DA |
| **Erythropoietin stimulating agents** | epoetin β, darbepoetin α | B03XA |

**Supplemental Table 3:** Characteristics of Sessions and Patients for Sensitivity Analysis Based on Exposure Definitions

| **Characteristics** | **Overall** | **Antihypertensive drug exposure** | | | | | |
| --- | --- | --- | --- | --- | --- | --- | --- |
|  |  | **ACEI/ARB** | **Alpha-blocker** | **Beta-blocker** | **DHP-CCB** | **No antiHTN tx** |  |
| Hemodialysis sessions, n (%)^a^ | 39371 (100%) | 2287 (6%) | 7302 (19%) | 9348 (24%) | 10866 (28%) | 23474 (60%) |  |
| Patient numbers, n (%)^b^ | 115 (100%) | 35 (30%) | 66 (57%) | 65 (57%) | 86 (75%) | 107 (93%) |  |
| Duration of included HD(days), mean (SD) ^d^ | 825.2 (261.1) | 752.3 (287.4) | 845.1 (221.0) | 853.4 (234.2) | 825.7 (246.8) | 846.8 (243.0) |  |
| Demographic data^a^ |  |  |  |  |  |  |  |
| Age (years), mean (SD) | 61.4 (15.1) | 57.8 (16.8) | 60.1 (15.3) | 59.4 (14.5) | 61.1 (15.9) | 61.3 (15.2) |  |
| Male, n (%) | 69 (60%) | 25 (71%) | 43 (65%) | 35 (54%) | 53 (62%) | 63 (59%) |  |
| Coronary artery disease, n (%) ^d^ | 28 (24%) | 6 (17%) | 11 (17%) | 11 (17%) | 14 (16%) | 27 (25%) |  |
| Diabetes mellitus, n (%) ^d^ | 45 (39%) | 13 (37%) | 28 (42%) | 24 (37%) | 33 (38%) | 43 (40%) |  |
| Hypertension, n (%) ^d^ | 90 (78%) | 30 (86%) | 52 (79%) | 56 (86%) | 69 (80%) | 83 (78%) |  |
| Left ventricular ejection fraction, mean (SD) ^d^ | 64.1 (11.8) | 65.6 (8.8) | 62.7 (10.6) | 63.4 (11.1) | 64.8 (10.4) | 64.3 (11.8) |  |
| Dialysis-related variables^b^ |  |  |  |  |  |  |  |
| Dry weight (kg), mean (SD) | 59.6 (13.6) | 64.6 (14.0) | 61.3 (15.5) | 63.3 (16.0) | 59.3 (14.1) | 59.1 (12.4) |  |
| Post hemodialysis body weight (kg), mean (SD) | 61.7 (14.0) | 65.6 (14.3) | 63.1 (16.4) | 64.8 (16.1) | 61.0 (14.9) | 61.3 (12.6) |  |
| Ultrafiltration (kg), mean (SD) | 2.3 (1.2) | 2.2 (1.0) | 2.3 (1.2) | 2.5 (1.2) | 2.3 (1.1) | 2.4 (1.2) |  |
| UF_DW_percent(%), mean (SD) ^c^ | 4.0 (1.8) | 3.4 (1.6) | 3.9 (1.8) | 3.9 (1.7) | 3.9 (1.7) | 4.0 (1.9) |  |
| Blood flow (ml/min), mean (SD) | 269.1 (31.6) | 291.2 (31.0) | 280.5 (31.0) | 280.8 (32.6) | 277.6 (33.6) | 264.2 (29.5) |  |
| Dialysate flow (ml/min), mean (SD) | 585.4 (129.0) | 675.5 (136.4) | 632.6 (141.1) | 627.6 (137.2) | 610.9 (138.2) | 569.6 (121.6) |  |
| Dialysate Avg. Temp (°C), mean (SD) | 36.1 (0.4) | 36.2 (0.4) | 36.1 (0.3) | 36.2 (0.4) | 36.2 (0.4) | 36.1 (0.3) |  |
| Normal saline (ml), mean (SD) | 513.8 (69.2) | 546.4 (139.9) | 523.8 (96.6) | 527.0 (96.6) | 521.2 (91.2) | 509.6 (55.4) |  |
| Pre-HD SBP (mmHg), mean (SD) ^b c^ | 136.0 (22.4) | 145.6 (19.8) | 143.0 (20.8) | 142.0 (20.4) | 143.7 (19.4) | 132.0 (22.9) |  |
| Pre-HD DBP (mmHg), mean (SD) ^c^ | 72.9 (14.4) | 75.0 (14.6) | 77.0 (15.4) | 75.1 (14.4) | 76.5 (14.3) | 71.2 (14.3) |  |
| Post-HD SBP (mmHg), mean (SD) ^c^ | 131.5 (28.6) | 152.6 (24.2) | 144.0 (25.6) | 141.0 (25.7) | 144.3 (25.1) | 125.2 (28.8) |  |
| Post-HD DBP (mmHg), mean (SD) ^c^ | 73.1 (17.0) | 79.5 (16.0) | 80.1 (20.0) | 76.1 (15.6) | 78.9 (17.6) | 70.5 (15.2) |  |
| Dialysate Calcium, n (%) |  |  |  |  |  |  |  |
| 2.5 mEq/L | 17061.0 (43%) | 1050.0 (46%) | 2632.0 (36%) | 3015.0 (32%) | 5043.0 (46%) | 10224 (44%) |  |
| 3 mEq/L | 18865 (48%) | 676 (30%) | 3698 (51%) | 4886 (52%) | 4561 (42%) | 11689 (50%) |  |
| 3.5 mEq/L | 3082 (8%) | 561 (25%) | 958 (13%) | 1420 (15%) | 1243 (11%) | 1239 (5%) |  |
| Co-medication, n (%)^bd^ |  |  |  |  |  |  |  |
| Midodrine | 8715 (22%) | 31 (1%) | 599 (8%) | 1321 (14%) | 439 (4%) | 7008 (30%) |  |
| Vasodilator | 2235 (6%) | 365 (16%) | 787 (11%) | 962 (10%) | 1308 (12%) | 606 (3%) |  |
| Erythropoietin | 33271 (85%) | 2181 (95%) | 6619 (91%) | 8106 (87%) | 9826 (90%) | 19286 (82%) |  |
| Average ambient temperature(°C), mean (SD) | 25.1 (4.3) | 25.5 (4.4) | 25.1 (4.4) | 25.1 (4.4) | 24.8 (4.5) | 25.2 (4.2) |  |

**^a^**Characteristics are presented in patient numbers.

**^b^**Characteristics are presented in sessions.

**^c^** UF_DW_percent: ultrafiltration divided by dry weight, pre-HD SBP: pre-hemodialysis systolic blood pressure, pre-HD DBP: pre-hemodialysis diastolic blood pressure

**^d^**Duration of included HD (days) was the difference between the earliest and latest session date of each patient included in our analysis; CAD: including a documented history of CAD, history of angina, previous myocardial infarction, previous revascularization procedures (CABG, PCI); LVEF: left ventricular ejection fraction was documented based on the echocardiograms date closest to each dialysis session; CAD, DM, HTN were considered affirmed if the patient had respective records during the study period; vasodilators including hydralazine, minoxidil, and nitrates; vasodilators and midodrine were identified if they were prescribed on the dates corresponding to each dialysis session; erythropoietin, including epoetin β, darbepoetin α, were accounted for if a prescription was given within the 7-day window surrounding each session, average ambient temperature, attained by each patient's address matched through GPS to the nearest weather station, and the average ambient temperature recorded during the 24 hours before each dialysis session was calculated.

**Supplemental Table 4:** Operational Definition in Sensitivity Analysis for Outcome Definition

| **Additional normal saline supplement** | At NCKU Hospital, the typical amount of normal saline used during a dialysis session for tube irrigation was between 300 and 400 mL. Thus, we reasoned that any saline supplement exceeding 600 mL was likely an additional supplement given in response to an IDH event |
| --- | --- |
| **Adjusted dialysis target** | Failure to reach the predetermined dry weight, characterized by a post-hemodialysis body weight exceeding the set dry weight. |
